# Expression of Toll-like receptors 7-10 in human fallopian tubes

**Published:** 2014-06

**Authors:** Nasrin Ghasemi, Fatemehsadat Amjadi, Ensieh Salehi, Mojgan Shakeri, Abbas Aflatoonian, Reza Aflatoonian

**Affiliations:** 1 *Research and Clinical Center for Infertility, Shahid Sadoughi University of Medical Sciences, Yazd, Iran.*; 2 *Department of Anatomy, Tehran University of Medical Sciences, Tehran, Iran.*; 3 *Tehran Islamic Azad University of Medical Sciences, Tehran, Iran.*; 4 *Department of Endocrinology and Female Infertility, Reproductive Biomedicine Research Center, Royan Institute for Reproductive Biomedicine, ACECR, Tehran, Iran.*

**Keywords:** *Fallopian tube*, *Innate im*munity, Toll like receptors (TLRs), Reproductive medicine

## Abstract

**Background:** The human female reproductive tract (FRT) is constantly deal with the invading pathogens. Recognition of these pathogens is attributed to the family of Toll like receptors (TLR) as a major part of the innate immune system. We and others have previously revealed that TLRs1-6 express in the female reproductive tract. However, more studies should be done to detect TLRs 7-10 in the female reproductive tract, especially in the fallopian tubes.

**Objective:** To examine the expression of TLRs7-10 in human fallopian tube tissue.

**Materials and Methods: **Using immunostaining techniques, distribution of TLR7-10 was studied in surgical sections from the uterine tubes, obtained from patients undergoing tubal ligation and hysterectomy for benign gynecological conditions. RT-PCR was used to show the existence of TLR7-10 genes in fallopian tube tissue.

**Results: **TLR7-10 proteins were detected in the fallopian tube epithelium, although the intensity of staining was not equal in cases. TLR7-10 genes were expressed in human fallopian tube tissue.

**Conclusion:** This study indicates that TLR7-10 is expressed in fallopian tubes tissues, and may play an important role in microbial recognition, and in host defense against ascending infection.

## Introduction

The human female reproductive tract (FRT) is constantly contact with the invading microorganisms and pathogens transmissible through intercourse which can ascend from the lower to the upper parts (1). Infectious and inflammatory disorder of the upper parts specially the fallopian tubes can have serious consequences such as tubal occlusion, chronic pelvic inflammation and infertility (2, 3). Moreover, increasing evidence suggests Chlamydia trachomatis infection predisposes the tubal microenvironment to ectopic implantation (4).

Characterization of the defense systems present within the female reproductive tract will assist the development of effective therapies or vaccination strategies against sexual transmitted diseases. The innate immune system is the first line of defence for the purpose of protection from pathogenic challenge and limiting related reproductive disorders (5, 6). Epithelial cells have evolved innate immune antimicrobial functions. Important mediators of microbial detection are germ line encoded receptors called Toll like receptors (TLR) (7). TLRs recognize pathogen-associated molecular patterns (PAMPs) and endogenous damage-associated molecular patterns (DAMPs) synthesized by microorganisms (8, 9). Until now, at least 10 human TLRs and 13 mouse TLRs have been described (10). 

TLRs 1-9 are conserved between human and mouse. TLR1, 2, 4, 5, 6 are located on the plasma membrane and detect pathogen membrane components while TLR3, 7, 8, 9 are expressed in cytoplasmic organelles (10, 11). Each individual TLR is known to detect molecules (ligands) from varying classes of microbial agents (6, 12). ''The recognition of bacterial PAMPs like LPS, PGN, flagellin is mediated by TLR1, 2, 4, 5 and 6. TLR3, 7, 8, 9 recognize nucleic acids. TLR7 and TLR8 detect nucleotide derivates, such as self and viral single-stranded RNA (ds RNA). In contrast, TLR3 recognizes double-stranded RNA. TLR9 binds bacterial un-methylated DNA'' (13). 

TLR10 is highly homologous to TLR2 and is probably another TLR2-associated receptor, but its function is still unknown and no specific ligand has yet been identified for TLR10 (14-16). Several studies have investigated the presence and the role of TLRs in the male and female reproductive tract (7, 8, 17-24). It is evident that TLRs are involved in pathophysiology of disorders like PCO, endometriosis and poor ovarian responder (25-27). However, little has been done to identify the expression of these receptors in fallopian tubes. Because of the key role TLRs play in mediating innate immune defense, the aim of the present study was to determine TLR7-10 expression and immunostaining localization in human fallopian tubes tissue. We examined this hypothesis and our results showed that TLR7-10 were present in the fallopian tubes tissue.

## Materials and methods

**Patients and samples**

In this cross sectional study, fallopian tube mucosal tissue was obtained following surgery from nine patients who underwent tubal ligation or hysterectomy for benign gynecological conditions. All the women taking part in the investigation showed no evidence of any pathological uterine disorder and had not used oral contraception or an intrauterine device in the previous 3 months. The average age of women taking part in this study was 44 (range 36-52) years. Small section of Fallopian tube tissue for immunostaining was immediately fixed in 10% formalin. For genomic studies, tissue sections from the same samples immediately placed in RNA later (Ambio, Huntingdon, U.K.) followed by immediate immersion in liquid nitrogen until processed. Approval to use tissues was prepared from the Local Ethics Committee and written informed consent was obtained prior to the collection of tissue samples.

**Antibodies and peptides**

Antibody and peptides used in the experiments were obtained from Santa Cruz Biotechnology (CA, USA). These were goat polyclonal antibodies specific for N-terminal domains of TLRs 7 and 9 (catalogue number sc13207 and sc13212, respectively), goat polyclonal antibody specific for V-terminal domains of TLR10 (catalogue number sc23577) and rabbit polyclonal specific for D-terminal domains of TLR8 (catalogue number sc13212-R). Blocking peptides specific for the respective antibodies were used to detect non-specific staining.

**Immunostaining**

Tissue samples were fixed in 10% neutral buffered formalin for 18-24h, embedded in paraffin and cut in 4μm and routinely stained with haematoxylin and eosin (H&E). Formalin-fixed sections were deparaffinized with xylosine twice for 5 min, followed by rehydration in graded ethanol. Endogenous peroxidase activity was blocked with3% v/v hydrogen peroxidase in methanol for 20 min. Antigen retrieval on these sections was performed by microwave irradiation for 10 min in 10 mmol/l sodium citrate pH= 6.0. Sections were allowed to cool for 20 min and then washed in PBS, then stained using a Vectastain Elite ABC peroxidase kit (Vector Laboratories Ltd, U.K.). 

In addition, to avoid non-specific binding, an avidin/biotin blocking kit (Vector) was used. Briefly, slides were blocked for 1 h at room temperature in PBS containing 0.2% v/v horse serum and 25% v/v avidin supplied in blocking kit. The block was removed and slides were incubated overnight at 4°C in primary antibody at an appropriate dilution using antibody diluent media (Dakocytomation Ltd, U.K.) containing 250 ml biotin per ml of diluted antibody. Binding was visualized by incubation with peroxidase substrate AEC (3-amino-9-ethylcarbazole) (Vector) for 10 min, washed in distilled water for 5 min and counterstained in 10% haematoxylin for 10 min. 

Slides were washed in tap water for 5 min and mounted with Aqua mount (VWR. Negative control wells were obtained by blocking of primary antibody with the corresponding specific peptide using a 20-fold excess of blocking peptide. Immunostained sections were examined using an Olympus BH2 microscope at ×250 magnification (Olympus, London, U.K.) (17).

**RT-PCR**

Tissues were removed from RNA later and homogenized in 1 ml of TRI reagent (Sigma, Pool, UK) using an Ultra Turrax homogenizer (VWR, Leicestershire, UK) for 2 min. Total RNA was extracted using TRI reagent standard protocol supplied by the manufacturer. Total RNA was extracted using TRI reagent standard protocol supplied by the manufacturer. Total RNA was treated with DNase I (Fermentas, Sanktleon-rot, Germany) to remove genomic DNA contamination from samples. 

First-strand cDNA synthesis was performed using oligo dT primers (Fermentas) and the Superscript II reverse transcriptase system (Invitrogen, Paisley, UK). Negative controls were prepared without addition of the enzyme (non-reverse transcribed controls, RT controls). The RT-PCR was performed by combining cDNA, Platinum Blue PCR Super Mix (Invitrogen) and the forward and reverse primers for TLR7-10 (Metabion, Martinsried, Germany). 

The used forward and reverse primer sequences are described in table I. The amplification was persistent for 40 cycles under the following setting; 95^o^C for 30 s, 59-65^o^C for 1min and 72^o^C for 2min (Table I). All experiments included RT controls as negative controls (no cDNA). 

To separate PCR products 10µl of each sample was resolved on a 1.2% agarose gel (Sigma) and electrophoresis was performed with 1x TAE buffer (Invitrogen) and a voltage of 95V for 30-40 min. The bands were visualized by using an ultraviolet trans illumination and digital images were captured by Gel documentary machine (Care stream, Berlin, Germany). The amplified PCR products were sequenced to confirm the identity of the amplified product.

## Results

**Immunostaining**

**Figure 1:**
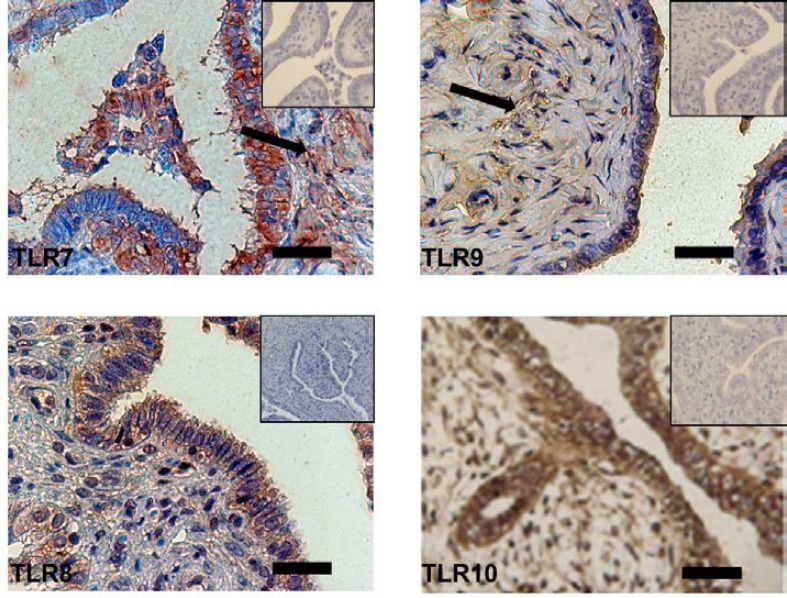
Immunostaining of Toll-like receptor (TLR) 7-10 expression in human fallopian tubes. Positive staining is red-brownish and negative staining is blue. Insets show blocking of the anti-TLR7-10 antibodies with its specific peptides. Arrows depict cells in fallopian tubes stroma with weak staining with TLR 7 and 9 antibodies

Positive immunostaining for TLRs 7-10 was observed in the fallopian tube epithelium. The intensity of staining was not equal in cases. Strong TLR9 staining was observed in the apical part of epithelial cells. In addition, weak staining of stroma was observed for TLR7and 9 but no staining in stroma for others. Immunodetection of TLRs was specific since blocking of the primary antibodies with their respective blocking peptides abolished the staining. This immunostaining localization TLRs 7-10 has been shown in figure 1 (A-D respectively).

**RT-PCR**

**Figure 2:**
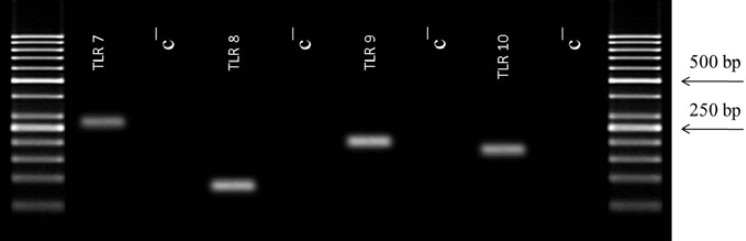
The expression of TLR7-10 genes obtained by RT-PCR in human fallopian tube tissue. Total RNA was extracted from fallopian tube and amplified with the primers. Each pair of primers produced a specific product with the specific predicted size in the test (T) samples. C= control samples

mRNA expression of TLR7-10 genes was detected in the epithelium of Fallopian tube. All amplified products were the expected size for that particular gene. There was no product amplified in control samples indicative of the lake of genomic DNA contamination. Figure 2 shows the result of RT-PCR for mRNA expression of TLR7-10 genes in the epithelium of Fallopian tube.

**Table I:** List of TLRs primer sequences used in the experiment for the amplification of human TLR 7-10 mRNA with RT-PCR

**Gene**	**Forward primer**	**Reverse primer**	**Annealing temperature** **(C)**	**Product size** **(bp)**
TLR7	CCTTGAGGCCAACAACATCT	GTAGGGACGGCTGTGACATT	63	285
TLR8	CTTCGATACCTAAACCTCTCTAGCAC	AAGATCCAGCACCTTCAGATGA	60	90
TLR9	TTCCCTGTAGCTGCTGTCC	ACAGCCAGTTGCAGTTCACC	60	207
TLR10	TGCCCACCACAATCTCTTCCATGA	AGCAGCTCGAAGGTTTGCCCA	60	184

## Discussion

TLR constitute a major part of the innate immune system (13). Considering their role in protecting the female reproductive tract against ascending pathogens, TLRs are expected to be present in these tissues. However, little study has been done to identify TLRs in the female reproductive tract particularly the fallopian tubes. Current investigation reports that human epithelial cells from the fallopian tube express TLR7-10 genes and proteins. Immune surveillance in the FRT is critically affected by interaction of many factors including hormones, resident leukocyte populations, and the distinct microenvironment of each component of the reproductive tract (28). 

In contrast to the lower portions, the upper regions of the FRT, notably the fallopian tubes have been considered sterile sites, emerging evidence shows that these regions of the FRT are subject to challenge by ascending pathogens, such as Neisseria gonorrhoeae and Chlamydia trachomatis (28). Clearance of Chlamydia trachomatis relys on detection of this microbe and triggers a series of alarm signals resulting in inflammatory immune response. The persistent of chlamydial infection leads to endosalpingeal tissue damage and tubal factor subfertility and may be related to pregnancy complications such as preeclampsia and preterm labor (29-31).

It is also well-documented that sperm and associated ejaculate can be the carrier for virus thereby exposing the upper reproductive tract to these pathogens (32, 33). Given the diversity of microbes to which the fallopian tubes is exposed, it is imperative that this tissue is responsive to pathogenic challenge. In this regard, TLRs function as sentinels that recognize microbial antigens and respond to a wide array of pathogens by rapidly initiating innate and adaptive immune responses. Our determination that human fallopian tubes express TLRs 7-10 is in agreement with the study conducted by Hart *et al* regarding the constitutive presence of TLRs7-10 in FRT tissues including Fallopian tubes, uterine endometrium, cervix and ectocervix (34). They have shown only genomic expression of TLRs 7-10 in oviduct tissues using RT-PCR while we also showed the localization of TLRs7-10 protein in the fallopian tube by immunostaining in addition to mRNA expression. 

In contrast to our findings, another experiment has demonstrated to express TLR1-9 but not 10 in FTEC (35). The explanation for the variance in these results could be differences between features of fallopian tube tissue and that in vitro study. Previous study from our laboratory has demonstrated expression of TLRs1-10 in epithelial cells of the human endometrium. It also have reported the presence of TLR9 in the sparse population of individual stromal cells (17). Now, we extend these findings to demonstrate constitutive expression of TLRs7-10 in the epithelium of fallopian tubes tissue. We have also observed weak staining for TLR9 in the stromal cells of fallopian tube. This variation could be beneficial for host defense against viral infections. However, it will be important to determine if these receptors are functional in the fallopian tubes after exposure to their ligands. 

Our previous observation demonstrated sex hormones effect on Toll-like receptors expression in endometrium during menstrual cycle, further investigation are needed to examine whether TLRs expression also illustrate cycle dependent pattern in the fallopian tubes (17, 36).

## Conclusion

In conclusion, our study demonstrates the in vivo localization of TLRs7-10 in the fallopian tubes of humans, modulating innate immunity in this region. However it merits further investigation to reveal how TLR function and expression is regulated in the fallopian tubes by sex hormones.
